# Fractional Flow Reserve Derived from Coronary Computed Tomography Angiography Datasets: The Next Frontier in Noninvasive Assessment of Coronary Artery Disease

**DOI:** 10.1155/2018/2680430

**Published:** 2018-07-25

**Authors:** Caroline Ball, Gianluca Pontone, Mark Rabbat

**Affiliations:** ^1^Loyola University Medical Center Department of Cardiology, Maywood, IL, USA; ^2^Centro Cardiologico Monzino, University of Milan, Italy

## Abstract

Fractional flow reserve (FFR) derived from coronary CTA datasets (FFR_CT_) is a major advance in cardiovascular imaging that provides critical information to the Heart Team without exposing the patient to excessive risk. Previously, invasive FFR measurements obtained during a cardiac catheterization have been demonstrated to reduce contrast use, number of stents, and cost of care and improve outcomes. However, there are barriers to routine use of FFR in the cardiac catheterization suite. FFR_CT_ values are obtained using resting 3D coronary CTA images using computational fluid dynamics. Several multicenter clinical trials have demonstrated the diagnostic superiority of FFR_CT_ over traditional coronary CTA for the diagnosis of functionally significant coronary artery disease. This review provides a background of FFR, technical aspects of FFR_CT_, clinical applications and interpretation of FFR_CT_ values, clinical trial data, and future directions of the technology.

## 1. Introduction

The last decade has brought rapid and exciting change to the field of cardiac imaging. In this context, coronary computed tomography angiography (CCTA) represents an excellent noninvasive tool for the evaluation of patients with coronary artery disease (CAD) [1–4]. The diagnostic accuracy of this technique has improved thanks to more effective strategies of premedication and implementation in image acquisition and postprocessing [5–10]. Likely, the most exciting technical advance is the ability to noninvasively measure the functional impact of coronary artery plaques. Advances in imaging techniques, mathematics, and computer science provide the ability to accurately measure fractional flow reserve (FFR) derived from coronary CTA datasets (FFR_CT_) [11]. FFR_CT_ has the ability to provide critical information to the Heart Team without exposing the patient to unnecessary risk of an invasive procedure. What follows is a review of FFR_CT_, including the theory and technology behind the imaging technique, accuracy data, clinical applications, and future directions.

## 2. Fractional Flow Reserve: Applications

**Figure 1:**
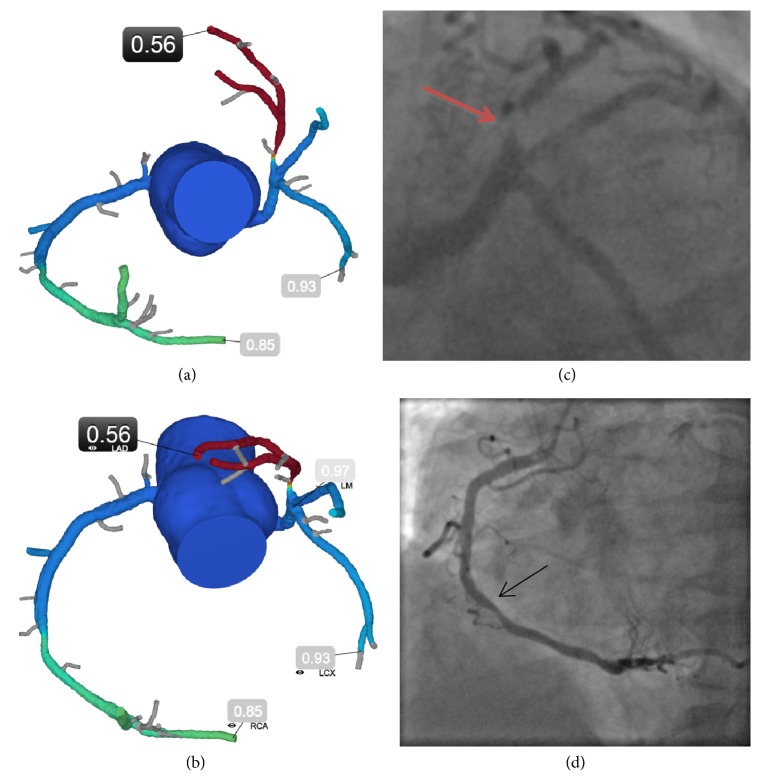
FFR_CT_ redefining revascularization strategy. A 68-year-old male with tobacco abuse, hypertension, hyperlipidemia, diabetes, and shortness of breath underwent coronary CTA demonstrating a 70%-90% stenosis of the proximal LAD and a 50%-70% stenosis of the mid-RCA. The initial decision based on the coronary anatomy alone was to refer the patient for coronary artery bypass graft surgery. However, FFR_CT_ was performed to help inform the invasive procedure. FFR_CT_ distal to the proximal LAD and mid-RCA stenoses were 0.56 and 0.85, respectively. The patient was rescheduled for PCI, received one stent in the proximal LAD, and is asymptomatic at three-year follow-up. Teaching points: with the functionally significant stenosis in the proximal LAD supplying a large territory of myocardium and his continued symptoms on optimal medical therapy, the patient was taken to the catheterization laboratory where a drug-eluting stent was placed. In addition, the cardiologist performed invasive FFR for the moderate stenosis in the RCA which was 0.86, corroborating the nonfunctionally significant lesion and no intervention was performed. This case highlights the unique opportunity to noninvasively provide physiological information on a per-lesion level. This enables a more informed decision around recommendations for ICA, specifically about which vessels to further interrogate and may redefine revascularization strategy. Even when the decision on referral to ICA is already taken because of symptoms and high-risk anatomy as determined by coronary CTA, FFR_CT_ may be of relevance by guiding decisions about other intermediate range lesions. FFR_CT_ (a,b) and ICA (c,d). LAD demonstrates a focal proximal severe stenosis (**red arrow**) that is hemodynamically significant. RCA demonstrates a focal mid moderate stenosis (**black arrow**) that is not hemodynamically significant. FFR_CT_ indicates fractional flow reserve derived from coronary computed tomography angiography (CTA) datasets; ICA, invasive coronary angiography; LAD, left anterior descending artery; and RCA, right coronary artery.

Traditionally, coronary artery plaques were identified via invasive coronary angiography (ICA), using visual assessment of vessel stenosis to determine when a patient required revascularization, regardless of whether the visual assessment findings were supported by quantitative coronary angiographic techniques. However, oftentimes this results in revascularizing lesions that are not hemodynamically significant or lesions that are not the true etiology of the patient's symptoms, as well as failing to identify hemodynamically significant lesions [12]. Several techniques are now available in the cardiac catheterization lab to assess the hemodynamic significance of coronary lesions and therefore guide the interventional cardiologist to appropriate revascularization. At present, the most widely accepted measure of the hemodynamic significance of coronary stenoses is fractional flow reserve (FFR) which serves to identify specific vessels and lesions that are prone to induce ischemia during appropriate stress. FFR is a measure of the ratio of maximal blood flow through the coronary artery distal to a stenotic lesion to the normal maximal blood flow. It is traditionally measured in the cardiac catheterization lab using a pressure wire and administering an intracoronary or intravenous vasodilator to produce maximal hyperemia [13]. For example, an FFR value of 0.75 means that a stenosis is causing a 25% drop in pressure across the lesion, which means that maximal hyperemic flow is equally reduced by 25%. Recent large trials have demonstrated the benefit of FFR as a tool to assess the appropriateness of revascularization, particularly for patients with stable coronary artery disease (CAD). The DEFER Trial demonstrated that it is safe to defer percutaneous coronary intervention (PCI) in patients with stable angina with lesions of >50% visual stenosis on ICA but an invasive FFR value ≥ 0.75 [14]. Further, the FAME I trial demonstrated that, in patients with stable multivessel CAD, using invasive FFR during PCI reduced a composite outcome of death, nonfatal myocardial infarction, and revascularization [15]. Moreover, the FAME investigators found a decreased use of contrast, fewer stents, and lower procedure-related costs in patients randomized to undergo FFR-guided revascularization. Among patients with stable CAD the FAME II trial found that PCI to lesions with an invasive FFR value ≤ 0.80 compared with optimal medical therapy reduced the composite outcome of death, nonfatal myocardial infarction, and urgent revascularization [16]. Five-year follow-up from the FAME II trial confirmed that an FFR-guided PCI strategy was associated with a significantly lower rate of a combined outcome of death, myocardial infarction, or urgent revascularization when compared to patients managed with medical therapy alone [17]. There is a continuous, inverse relationship between the numeric FFR value and adverse outcomes, which is true regardless of whether or not the lesion is revascularized [18]. Despite its demonstrated clinical benefit and recommendations by major societies such as the American College of Cardiology (class IIa recommendation) and the European Society of Cardiology (class IA recommendation), given the invasive nature of the FFR procedure, the added time, radiation, contrast administration and cost of adenosine which must be given to patients during FFR measurement, the high costs of the pressure-sensing wires, and limited reimbursement, FFR evaluation is infrequently performed in clinical practice [19–22]. Invasive FFR was performed in only 6.1% of patients using data from over 60,000 ICA cases in the American College of Cardiology registry [23]. A priori knowledge of the presence and functional significance of specific coronary artery lesions before angiography may aid the cardiologist in deciding whether or not to proceed with ICA and redefine the revascularization strategy (Figure 1).

## 3. Technical Aspects of FFR_CT_

Advances in computational fluid dynamics (CFD) allow determination of coronary flow from static high quality coronary CTA images. CFD is based on the Navier-Stokes equations [24, 25]. While the Newtonian laws of motion and the understanding of viscous fluid dynamics that underpin the Navier-Stokes equations have been used in other disciplines for centuries, it was not until recent advances in supercomputing that these equations could be applied to the complex, three-dimensional, and time-sensitive flow patterns of the coronary arteries.

CFD requires defining the vessel shape anatomically, as well as adequate descriptions of the “boundary conditions” of the arterial system. To define vessel shape for CFD calculations one must first obtain coronary CTA images in accordance with the Society of Cardiovascular Computed Tomography (SCCT) guidelines to sufficiently define the vessel walls [26]. Currently, the only commercially available mechanism for computing FFR_CT_ is via HeartFlow (HeartFlow Inc., Redwood, CA). For FFR_CT_, 3-dimensional (3D) geometric modelling and computationally intricate blood flow analysis require off-site supercomputing power, and boundary conditions are determined by allometric scaling laws and assumptions regarding microvascular resistance [25]. Computation of FFR_CT_ involves (a) construction of an accurate patient-specific 3D anatomic model of the epicardial coronaries, (b) specifying microcirculatory models for coronary blood flow during maximal hyperemia, and (c) performing a computational solution of the laws of physics governing fluid dynamics. The physiologic model is created using the patient's anatomical model and is based on 3 scientific principles: (1) resting coronary blood flow is quantified relative to the myocardial mass. Mass can be calculated from myocardial volume, which is easily extracted from volumetric CCTA data; (2) microcirculatory resistance at rest is inversely proportional to the size of the lumen; and (3) vasodilatory response of the coronary microvasculature to adenosine is predictable. The reproducibility of FFR_CT_ is high. In one study, the difference between the first and second FFR_CT_ analyses was 0.035 and for invasive FFR repeated measurements was 0.043 [27].

There is growing data evaluating the diagnostic performance of reduced order models and 1D processing of the image data without the use of supercomputers for coronary CTA-derived FFR [28–31]. These algorithms are not commercially available, will require more extensive testing prior to clinical use, and require approximately 1 hour of physician work effort to produce the anatomical models needed.

Advances in technology have reduced the total radiation exposure from CCTA, which results in lower radiation exposure in patients undergoing FFR_CT_. Some centers report performing CCTA at doses < 0.1mSv. [32]

## 4. Clinical Applications and Interpretation of FFR_CT_ Values

HeartFlow FFR_CT_ has been approved by the United States Food and Drug Administration (FDA) for functional evaluation of CAD and is currently commercially available. Recently, the NICE (National Institute for Health and Care Excellence) updated their chest pain guidelines which recommend coronary CTA as the initial diagnostic test for patients with stable chest pain and suspected CAD and issued positive medical guidance on FFR_CT_ stating the technology is safe, has high accuracy, and may avoid the need for ICA and reduce cost to the healthcare system [33, 34]. In clinical practice, the application of FFR_CT_ is to safely eliminate unnecessary ICA and to better identify patients who may benefit from revascularization [35]. In the most recent Appropriate Use Criteria (AUC) for coronary revascularization, the American College of Cardiology recognized FFR_CT_ as a noninvasive “combination technique” with coronary CTA to help guide treatment [36].

Currently, clinicians using FFR_CT_ provided an interactive color-coded 3D model of the coronary tree with FFR values reported distal to stenoses [37]. The physician can manipulate the interactive model, examine each coronary segment and vessel, and determine the location and severity of lesions along the length of the coronary artery. The primary role of FFR_CT_ both clinically and as evaluated in clinical trials is to act as an alternative to invasive FFR by evaluating the FFR_CT_ distal to a focal stenosis. Diffuse coronary artery disease without a focal stenosis may lead to a progressive pressure drop along the length of the vessel and the treatment of these patients warrants further investigation. Nadir FFR_CT_ values should not be used alone when determining the need for ICA or revascularization [38]. Clinical decision-making should involve additional information such as patient history, medication use, anatomy, location of stenoses, vessel size, and suitability for revascularization. Ongoing prospective clinical registries such as ADVANCE (Assessing Diagnostic Value of Noninvasive FFR_CT_ in Coronary Care) will shed light on the optimal treatment strategy for patients with diffuse CAD and progressive FFR_CT_ drop and which parameter (distal vessel tip value versus value distal to a lesion) is more appropriate to guide decision-making and yield superior prognostic information [39]. Of note, FFR_CT_ data were analyzed in 952 of the initial 1000 patients (95.2%) enrolled in the ADVANCE real-world registry [40].

## 5. FFR_CT_ Clinical Trials

To date, several multicenter clinical trials of FFR_CT_ have been completed and are summarized in Table 1 [41–44]. In the three large diagnostic accuracy studies comparing FFR_CT_ and coronary CTA to invasive FFR as the reference standard, FFR_CT_ had better diagnostic performance than coronary CTA alone [41–43]. The NXT (Analysis of Coronary Blood Flow Using CT Angiography: Next Steps) [45] trial is the latest diagnostic performance trial of FFR_CT_, which used the latest version 1.4 of the HeartFlow software. It was a 10-center prospective study and enrolled 254 patients and 484 vessels that were scheduled to undergo ICA for suspected stable CAD. Patients underwent coronary CTA and FFR_CT_ prior to the planned ICA. The investigators found an increased area under receiver-operating characteristic curve for FFR_CT_ (0.90, 95% CI 0.87-0.94) versus standard coronary CTA (0.81, 95% CI 0.76-0.87), which was statistically significant. Moreover, reported per-vessel sensitivities and specificities were 84% and 86%, respectively [43].

The PLATFORM (Prospective Longitudinal Trial of FFRCT: Outcome and Resource Impacts) study was a large multicenter prospective clinical utility trial of FFR_CT_ to assess clinical outcomes and sought to assess how FFR_CT_ affects the need for ICA [44]. The PLATFORM study assigned patients with new symptoms of stable ischemic heart disease to either “usual testing” or a coronary CTA/FFR_CT_-driven strategy. For patients in the planned invasive cohort, they either went directly to ICA or were assigned to a coronary CTA/FFR_CT_ strategy, with possible cancellation of the planned ICA based on the results of the coronary CTA/FFR_CT_. In the invasive arm of the PLATFORM study, a coronary CTA/FFR_CT_ strategy resulted in cancellation of 61% of previously planned ICA without any subjects with ICA cancelled experiencing an adverse event in 1-year follow-up. The use of a combined coronary CTA and FFR_CT_ strategy resulted in a reduction in the incidence of ICA showing nonobstructive disease by 83%. Importantly, follow-up at one year demonstrated lower health care costs for those patients in the planned invasive arm who underwent FFR_CT_ prior to ICA [44, 46, 47].

The FFR_CT_ RIPCORD study evaluated the impact of FFR_CT_ on clinical decision-making and demonstrated that the availability of FFR_CT_ results had a substantial effect on the labeling of significant CAD and management of patients compared to coronary CTA alone [48]. Data from 200 consecutive patients from the NXT trial were utilized. Three experienced cardiologists interpreted the coronary CTA data alone and reached a consensus on management strategy. FFR_CT_ data were then revealed to the same cardiologists and a second plan for each patient was again reached by consensus. FFR_CT_ resulted in a change in treatment decisions in 44% of patients. 30% of patients originally thought to require PCI based upon coronary CTA alone were reallocated to optimal medical therapy on the basis of a negative FFR_CT_. In fact, FFR_CT_ was >0.80 in 13 of 44 vessels (29.5%) graded as having a stenosis >90%. In contrast, FFR_CT_ was ≤0.80 in 17 of 366 vessels (4.6%) graded as having stenosis ≤50% [48]. These data and others underscore the unreliable relationship between anatomic measures of stenosis and lesion-specific ischemia [49, 50].

Clinical experience from Aarhus University Hospital demonstrated that deferring ICA in patients with FFR_CT_ >0.80 had favorable short-term prognosis (median follow-up period of 12 months) and was associated with a high rate of cancellation of planned ICA [51, 52].

## 6. Future Directions

The scope of FFR_CT_ reaches far beyond the identification of FFR values [53]. New measures, such as percent myocardium at risk, are on the horizon which should further help clinicians make decisions, especially about the clinical significance of distal or branch vessel stenosis. It is conceivable that revascularization in patients with small areas of ischemic myocardium, as determined by FFR_CT_, offers no advantage to optimal medical therapy alone. With the clinical adoption of FFR_CT_, we are seeing individuals with diffuse atherosclerosis and/or small coronary arteries with low FFR_CT_ values. These findings are in line with prior reports on the continuous decline in pressure along the length of diffuse atherosclerosis without focal stenosis [54]. The ratio of vascular volume to myocardial mass (V/M) may shed light into the ischemic potential of these patients and better characterize the disease states in patients with vessel sizes that are insufficient to meet myocardial demand, with or without focal stenoses [55].

Akin to the invasive arena, we may soon be able to utilize the anatomic and functional information derived from FFR_CT_ to calculate SYNTAX (Synergy between PCI with Taxus and Cardiac Surgery) scores to aid clinicians to both decide between optimal medical therapy and revascularization and between PCI and coronary artery bypass graft surgery. Recently, the calculation of the noninvasive functional SYNTAX score utilizing FFR_CT_ was noted to be feasible, yielded similar results to those obtained invasively, and reclassified 30% of patients from the high- and intermediate- SYNTAX score to the low-risk tertile (REF). [56] Applications of coronary CTA-derived computational models may enable us to determine outcomes after revascularization. Virtual stenting by FFR_CT_ demonstrated diagnostic accuracy of 96% in the prediction of residual lesions prone to ischemia when placed under the appropriate stress [57]. Virtual stenting and bypass grafting have the potential to advance our knowledge and optimize coronary revascularization. Finally, data from the EMERALD (exploring the mechanism of the plaque rupture in acute coronary syndrome [ACS] using CCTA and CFD) study illustrated that CFD derived hemodynamic forces across lesions improved the prediction of acute coronary syndrome [58]. In fact, noninvasive hemodynamic parameters were better at identifying culprit lesions causal of acute coronary syndrome than either stenosis severity or high-risk plaque features. This data demonstrates the extraordinary potential bridging CFD to coronary CTA to provide not just an FFR value but valuable insight into identifying the* vulnerable patient*.

## 7. Conclusion

FFR_CT_ represents an exciting development in the evaluation of ischemic heart disease. Using advances in imaging and CFD, FFR_CT_ offers a noninvasive diagnostic strategy to identify functionally significant lesions in order to distinguish between patients who can safely avoid ICA and those patients who require revascularization.

## Figures and Tables

**Table 1:** Summary of presented FFR_CT_ clinical trials.

Trial	Study Population	n	Intervention	Findings
NXT	Stable CAD scheduled to undergo invasive angiography	251	CCTA vs FFR_CT_	FFR_CT_ had higher diagnostic accuracy than CCTA

PLATFORM	New stable CAD	584	Noninvasive stress testing vs FFR_CT_ and ICA vs FFR_CT_ prior to ICA	In patients randomized to an early invasive coronary angiogram for stable CAD, FFR_CT_ was associated with a lower rate of angiography showing no obstructive CAD and safe cancellation of ICA.

RIPCORD	Stable chest pain	200	CTA vs FFR_CT_	FFR_CT_ data resulted in a change in management in 36% of cases.

ADVANCE	Stable CAD	1000	CCTA Findings Reviewed	CCTA stenosis severity, importantly, even for mild CCTA stenosis, in addition to diabetes and hypertension were predictive of abnormal FFR_CT_.

Functional Syntax Score	Stable multivessel disease	77	Noninvasive vs invasive anatomic and functional SYNTAX score.	Functional SYNTAX score utilizing FFR_CT_ yielded similar results to those obtained invasively and reclassified 30% of patients from the high- and intermediate- SYNTAX score to the low-risk tertile. FFR_CT_ has good accuracy in detecting functionally significant lesions in patients with multivessel disease.

CAD = coronary artery disease. CCTA = coronary computed tomography angiography. FFR_CT_ = CTA-derived fractional flow reserve. ICA = invasive coronary angiography.

## Abbreviations

CCTA: Coronary computed tomography angiography
FFR: Fractional flow reserve
FFR_CT_: Fractional flow reserve derived from coronary computed tomography angiography datasets
ICA: Invasive coronary angiography
PCI: Percutaneous coronary intervention
CFD: Computational fluid dynamics
FDA: Food and Drug Administration
CAD: Coronary artery disease.
